# Rearing zombie flies: Laboratory culturing of the behaviourally manipulating fungal pathogen *Entomophthora muscae*

**DOI:** 10.1016/j.mex.2023.102523

**Published:** 2023-12-13

**Authors:** Sam Edwards, Henrik H. De Fine Licht

**Affiliations:** a Department of Plant and Environmental Sciences, University of Copenhagen, Thorvaldsensvej 40, Frederiksberg C. 1871, Denmark; b Living Systems Institute, Biosciencesv, University of Exeter, Exeter EX4 4QD, United Kingdom

**Keywords:** Insect pathogenic fungus, *Diptera*, Host-parasite interaction, Behavioural manipulation model, *In vivo*, *In vitro*, Isolation and culturing of Entomophthora muscae

## Abstract

Insect pathogenic fungi (IPF) and insects have ubiquitous interactions in nature. The extent of these interkingdom host-pathogen interactions are both complex and diverse. Some IPF, notably of the order Entomophthorales, manipulate their species-specific host before death. The fungus-induced altered insect behaviours are sequential and can accurately be repeatedly characterised temporally, making them a valuable model for understanding the molecular and chemical underpinnings of behaviour and host-pathogen co-evolutionary biology. Here, we present methods for the isolation and laboratory culturing of the emerging behaviourally manipulating model IPF *Entomophthora muscae* for experimentation.

• *E. muscae* isolation and culturing *in vitro*.
• Establishing and maintaining an *E. muscae* culture *in vivo* in houseflies (*Musca domestica*).
• Controlled *E. muscae* infections for virulence experiments and quantification of conidia discharge per cadaver.

## Graphical abstract

**Figure:**
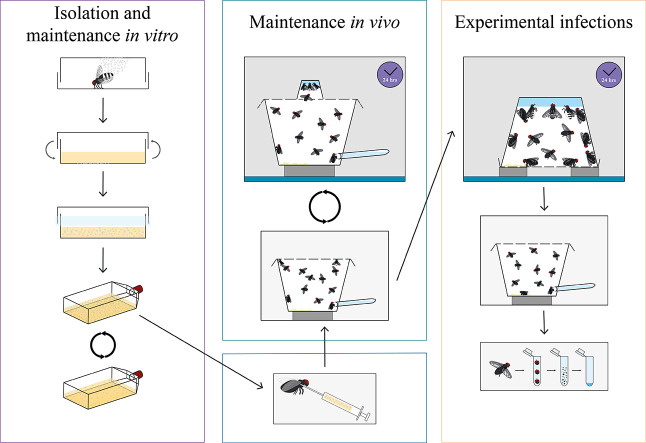

Specifications table

**Table:** 

Subject area:	Agricultural and Biological Sciences
More specific subject area:	Entomopathogenic fungi
Name of your method:	Isolation and culturing of Entomophthora muscae
Name and reference of original method:	N/A
Resource availability:	Resources are included in the text.

**Method details**

## Background

Houseflies are one of the most widespread species of insect in the world [1,2]. In part due to their global distribution, this resilient species is easily reared *en masse* for animal food and feed, and for waste management [3], [4], [5]. Additionally, being vectors of over 130 human and animal food-borne diseases, there has been interest in using natural pathogens as control agents [6]. The obligate entomopathogenic fungus *Entomophthora muscae* [7], has been explored as a biological control agent due to its high-host specificity [8], [9], [10]. The fungus *E. muscae* has been reported to infect up to a 100% of housefly populations in the wild, being particularly prolific in semi-closed environments of high fly density, such as in byres [11].

**Fig. 1:**
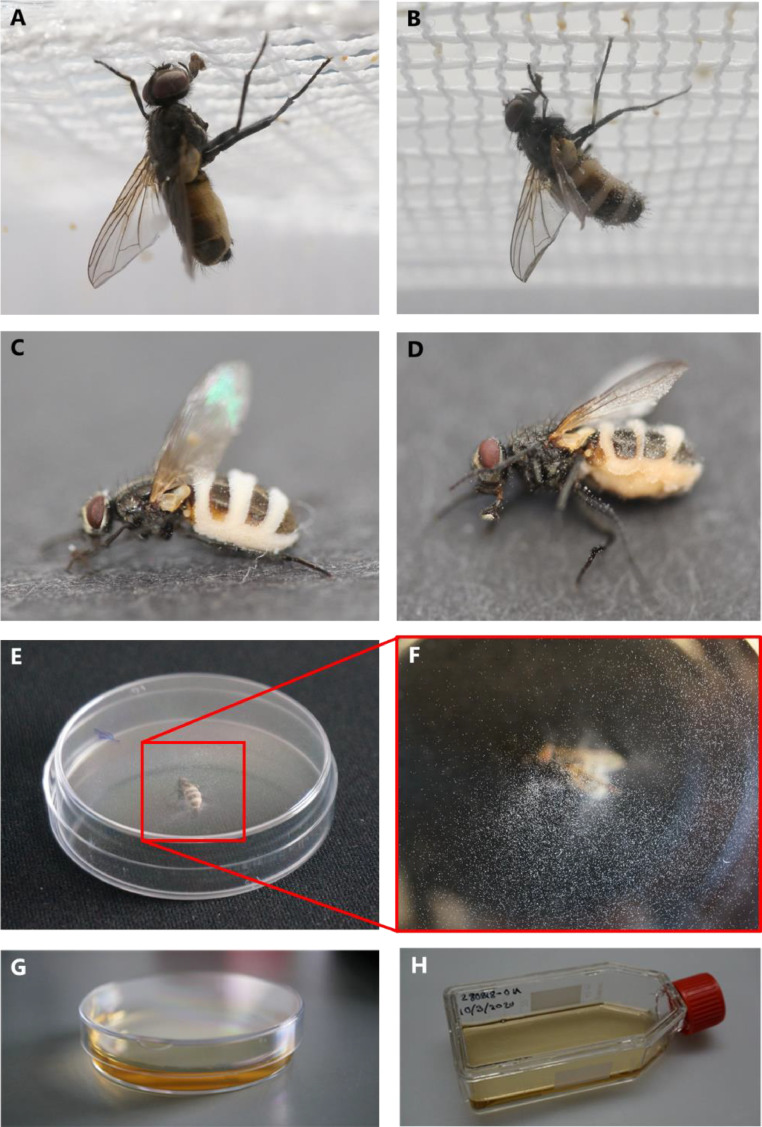
Isolation *in vitro* of *Entomophthora muscae* from a dead housefly cadaver. (A) A housefly newly killed by *E. muscae* (within one hour of death). The fly has been forced to summit to die at an elevated position, its proboscis is extended and wings are raised, and the fungus has not yet begun to grow out of the soft intersegmental membrane yet. (B).Four to eight hours after death, the fungus has penetrated through the intersegmental membrane and conidia have begun to be discharged (opaque clumps on abdominal hairs). (C) Flies collected four to eight hours, and (D) ca. 16 h after death. With time, the cadavers darken in colour and have more conidia present on the wings and body of the dead fly. (E) The cadaver is placed with its back towards the roof of a ‘downside-up’ upside-down petri dish. (F) Insert shows visible conidia on the roof of the ‘downside-up’ petri dish. (G) The petri dish is righted and half-filled with liquid media then sealed with Parafilm® then left for 2–6 weeks to check for growth, (H) the growing culture is transferred to a cell culture flask for long-term growth and storage. Photos: S. Edwards.

Following exposure of houseflies to *E. muscae* spores (conidia), the spores will germinate and penetrate the cuticle of the fly to gain access to the hemocoel [12]. In houseflies, the within-host life cycle of *E. muscae* occurs over six to seven days, during which time the fungus proliferates logistically as wall-less cells (protoplasts) considered to help the fungus evade the host's immune system [13]. During this time, *E. muscae*-infected houseflies exhibit reduced activity, altered flight patterns, and reduced reproductive capabilities [14,15]. Towards the end of the within-host stage of infection, infected flies are forced by the fungus to climb to an elevated position, extend and affix their proboscides to the substrate surface, and raise their wings as the host dies (Fig. 1A). One of the peculiarities of this insect pathogenic fungus is that the apparent obligate behavioural manipulation of the moribund host prior to death occurs within four hours before sunset [16,17]. This predictable timing of the behavioural manipulation onset makes this an excellent system for unravelling the mechanism behind the behavioural manipulation phenomenon [16].

A couple of hours after host death, the fungus emerges from between the intersegmental membrane of the host abdomen (Fig. 1B–D) [16,17]. The active discharge of infectious spores into the environment (Sporulation) occurs over ca. 24 h, whereby the infectious cadavers start to desiccate and lose infectivity (Fig. 1B–D). The conidia are forcibly ejected from conidiophores at high speed to a considerable distance to be horizontally transmitted to other healthy conspecifics [18,19]. Fungal isolates within the species complex of *E. muscae* have been found to be host-specific, with specific isolates only infecting a single host species naturally [16,20,21], although under laboratory conditions it is possible to cross-infect other host species, including *Drosophila melanogaster* [22] (for greater detail about the biology of *E. muscae*, please refer to references [16,20 and 23]). The genetic underpinnings of many of these unique traits are beginning to be unravelled due to genome sequencing efforts enabling studies of ‘omics methods to investigate the molecular basis for interactions between *Entomophthora muscae* and its dipteran hosts [24].

While *E. muscae* is commonly found in many areas [16,23], the difficulty with which it can be isolated and the usually slow *in vitro* growth of fungal cultures have hampered widespread research progress [25]. Here we present protocols for how to isolate and maintain *E. muscae*, both *in vivo* and *in vitro*. We also provide specific protocols on how to perform infections for experimental procedures, which provide up to 100% infection and mortality in our study system.

## E. muscae isolation and culturing *in vitro*

This protocol is designed to acquire *in vitro* isolation of the fungus from dead sporulating cadavers (from the laboratory or collected in the field) for applications like genomic DNA or RNA extraction and culturing of the fungal pathogen *in vitro* (Fig. 1) [25]. To obtain a liquid culture, *E. muscae* can be cultured in GLEN or Grace's Insect media (Tables 1, 2). Growth of the liquid cultures are slow and usually take 2–6 weeks.

**Table 1:** GLEN medium [25], [26], [27]. Recipe below is for 1 litre of medium. Adjust base medium (ingredients without fetal bovine serum (FBS)) to pH 7.0 with 1 N NaOH before autoclaving. *Important note*: Always add FBS after all other ingredients are autoclaved or filter sterilised. FBS can be used at a final concentration of between 5 and 10% and the amount of distilled water should be adjusted accordingly.

Ingredients	Quantity
Distilled water	900–950 mL
Glucose	4 g
Yeast extract	5 g
Lactalbumin hydrolysate	6.5 g
nacl	7.7 g
Fetal bovine serum (∼5–10%)	50–100 ml
Optional: MES (2-[N-*morpholino*]ethane sulfonic acid] buffer (Sigma-Aldrich, M-8250)	1.952 g

**Table 2:** Grace's Insect Medium (Sigma-Aldrich, G9771). Recipe below is for 1 litre of medium. This medium is available commercially and is usually supplemented with 5% (some laboratories use 10% to 20%) fetal bovine serum (FBS). Remember to adjust amount Grace's Insect Medium accordingly. *Important note*: Always add FBS after all other ingredients are autoclaved or filter sterilised. The Agricultural Research Service Collection of Entomopathogenic Fungal Cultures (ARSEF) uses Grace's Insect Medium for entomophthoralean culture. The ARSEF is the world's largest collection of living invertebrate pathogenic and associated fungi, and provide taxonomic databases and information, as well as useful information such as solid and liquid media recipes (https://www.ars.usda.gov/ARSUserFiles/80620520/media_recipes.pdf) [28].

Ingredients	Quantity
Grace's insect medium (sigma-aldrich, g9771)	800–950 ml
Fetal bovine serum (5–20%)	50–200 ml

### Materials

• Dead fly cadavers sporulating with *E. muscae* (ideally 6–18 h old) (Fig. 1B–D).
• Petri dishes, sterile.
• Liquid culture medium (e.g. GLEN, Grace's Insect Medium; Tables 1,2).
• Sterile 10 or 25 mL pipette tips and pipette.
• Parafilm®.
• 50 mL cell culture flask (e.g. Greiner Bio-One CELLSTAR®, Germany).
  1. Place a sporulating cadaver (Fig. 1B-D) (removal of wings will decrease obstruction to conidial distribution, but not essential) in the lid of a ‘downside-up’ petri dish [25], keeping the dish bottom (hereafter called ‘upper part’ due to it being upside down) untouched and sterile (Fig. 1E). Ensure the sporulating cadaver is positioned so that the actively discharged conidia can reach the upper part of the petri dish, this often means placing the fly cadaver with the dorsal side downwards.
  2. Leave for 30 min minimum to allow the cadaver's conidia to eject and stick to the surface of the upper part. Exact duration will vary based on the stage of conidiophore maturation and amount of conidia being discharged, best option is to check conidial quantity before proceeding to step 3. Anything between 30 min and 8 h have worked in our experience. However, we also experienced that the longer you leave the cadaver to sporulate, the higher the chance of the culture being contaminated (possibly from contaminants on the fly itself). Conidia should be visible on the underside of the upper-part of the petri dish before proceeding to step 3 (Fig. 1F).
  3. Remove upper part with conidia and place with a new sterile lid. Turn so the petri dish is placed normally with the lid on top. Add liquid media enough to cover the entire surface of the petri dish bottom in a layer ca. half the height of the petri dish (amount of liquid depends on the size of petri dish used) (Fig. 1G).
  4. Seal the petri dish with Parafilm® and leave at room temperature ca. 21°C or at 18°C depending on habitat where the *E. muscae* naturally occurs and away from light until growth is visible. Check once every week for proliferation from the conidia of *E. muscae* cells growing in the media using an inverted microscope (see examples of *E. muscae* cell morphology *in-vitro* in [27,29]. May take 2–6 weeks before growth is visible.
  5. When growth is visible and aplenty, transfer the growing culture to a cell culture flask and supplement with 5 mL of fresh liquid media (Fig. 1H).
  6. Repeat step 5 every 4–6 weeks, adding 10 mL of media to 1 mL of liquid culture in new cell culture flasks. See example in Fig. 7A&B of Elya and De Fine Licht [16], for assessing the health of the growing culture. Different isolates of *E. muscae* may grow differently *in vitro*, and more than one type of fungal cell morphology [27,29] may be present at the same time in a growing culture.

## E. muscae culture maintenance *in vivo* in houseflies (Musca domestica)

**Fig. 2:**
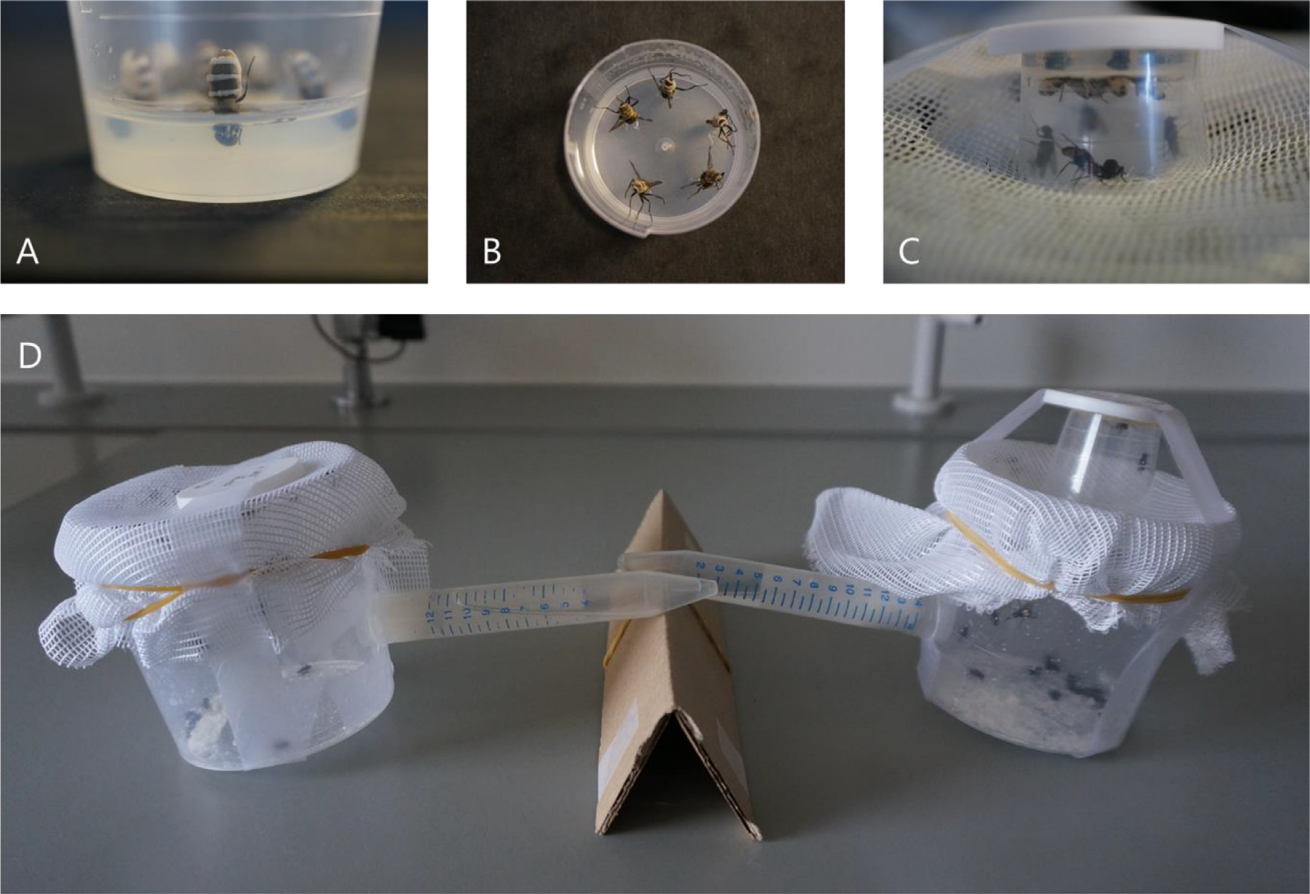
*In vivo* maintenance of *E. muscae* by placing sporulating cadavers atop a housing cup so that conidia rain down upon the healthy flies below. (A) Cadavers are placed head first into water agar with abdomens exposed for conidia release. (B) Multiple cadavers, here five, can be arranged in the agar for infection. (C) Flies can investigate the sporulating cadavers with access through the hole in the net. (D) Housing containers at a slight elevated angle by resting the falcon tube on a cardboard support. Left container: uninfected houseflies in a maintenance container (as in step 1 of this protocol). Right container: The medicine cup of cadavers in tapped upside down on the housing container. Both containers have food and water supplied ad libitum. Photos: S. Edwards.

This protocol is designed for in vivo maintenance of *E. muscae* in live laboratory-maintained houseflies. The number of cadavers used to infect a number of healthy conspecifics will create temporal variation for future cadaver collection, but this will still usually fall within 5 to 8 days after initial exposure. The maintenance of this system strongly relies on the maintenance of a housefly system, as they will be the future cadavers used for experiments or continuation of the system *in vivo* (Fig. 2). Infections are thus easily accomplished by using fresh sporulating cadavers (0–18 h following death of the host), which can be refrigerated for a few days at 5 °C and subsequently be used for infections at a later date, although this may lower virulence. Host death and sporulation can also be delayed by leaving infected flies (three to five days post infection) at 5 °C for a few days, this delays fungal progression and artificially extends the within-host life cycle of the fungus.

### Materials

• Dead sporulating cadavers (use the same fungal isolate) –– one to six cadavers is sufficient.
• 30 mL medicine cup containing 2–5 mL of 1.5% water agar.
• Entomology forceps.
• Humid chamber – made up of a plastic box containing water soaked paper towels.
• 18–23 °C incubator or room on a light:dark cycle between 16:8 and 12:12.
• Clear tape.
• Elastic bands.
• Netted mesh (20 × 20 cm) with a hole (1 × 1 cm in the centre).
• 365 mL (8.5 × 8 cm) plastic honey cups with a circular hole the diameter of a 15 mL falcon tube in the side, the hole needs to be made.
• 15 mL falcon tube filled with distilled water and plugged with cotton.
• Food – 1:1 ratio of skimmed milk powder and caster suga.r
• CO2 (carbon dioxide).
  1. Houseflies are housed in a plastic honey cup containing ad libitum food and water. Water is available from a falcon tub filled with demineralised water and plugged with cotton inserted into the hole in the pot side. The cup is covered by the netted mesh and held in place by elastic bands. A medicine cup lid is placed over the hole in the net and maintained in place with clear tape to prevent flies from escaping. To ensure continued access to water, we place containers at a slight elevated angle (e.g. resting the falcon tube on a cardboard support) so the water in the falcon tube is covering the cotton lid (Fig. 2D).
  2. Using cleaned forceps, gently grab a cadaver by the head and thorax, and bury the head and thorax into the agar in a medicine cup, keeping the abdomen exposed (Fig. 2A, B). Poking a hole in the agar beforehand with the forceps will reduce the risk of decapitating the cadaver.
  3. After placing one to six (or more) cadavers in this manner, place the medicine cup upside down and over the hole cut in the netted lid covering the housefly container (Fig. 2C). The live flies can optionally be anaesthetised using CO2 (carbon dioxide) to simplify this.
  4. Fix the medicine cup in place using clear tape, using the medicine cup lid as a label for identification.
  5. Place the container in a humid chamber for 24 h to allow for optimal sporulation conditions.
  6. After 24 h, remove the containers and keep in fixed temperature and light:dark conditions.

## Establishing an *in vivo E. muscae* culture in houseflies by injection

**Fig. 3:**
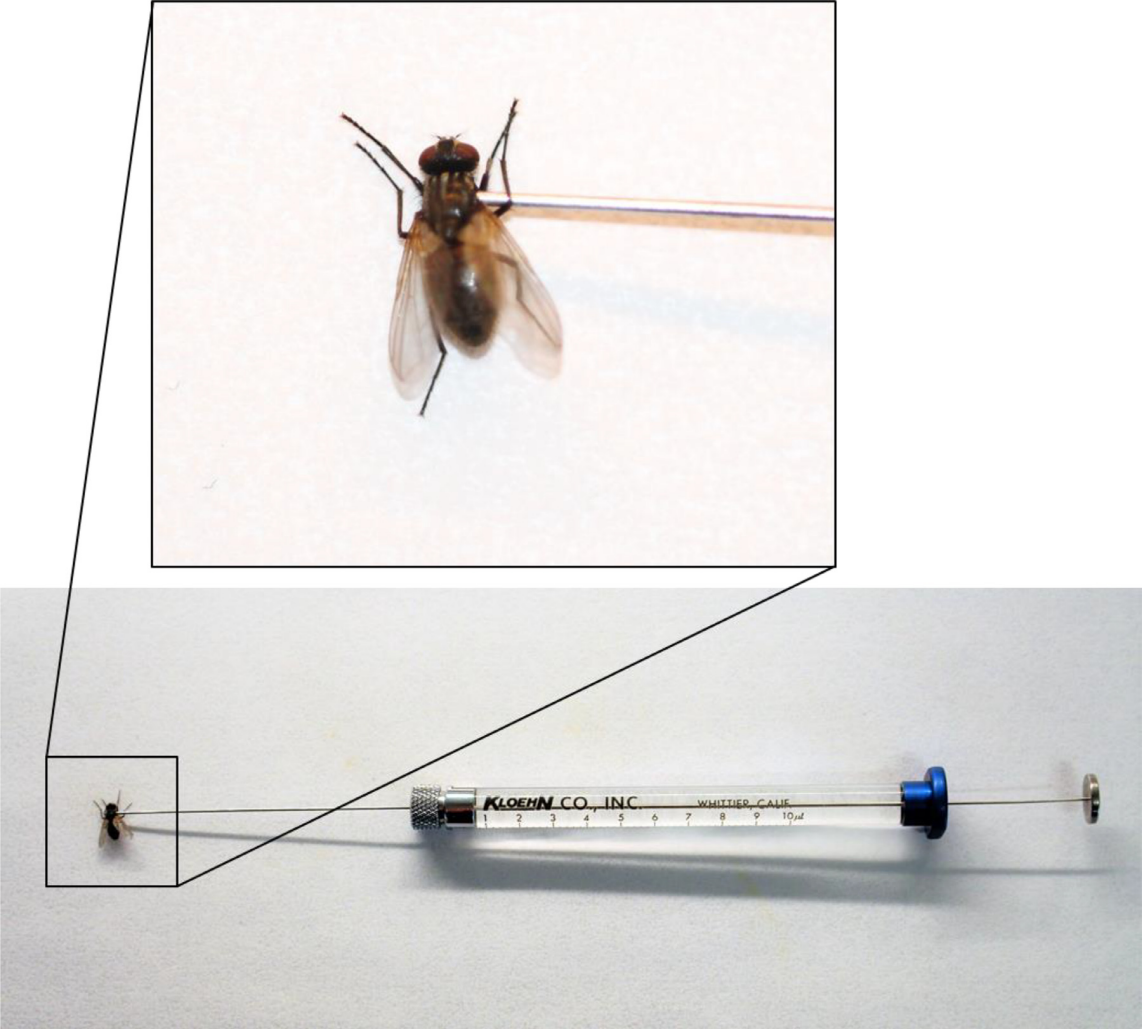
Injections are done with 1–3 µL of *E. muscae* liquid culture into the thorax of the houseflies. Insert shows a close-up of the syringe needle in a female housefly's thorax. Photos: S. Edwards.

This protocol is designed to transfer a liquid culture of *E. muscae* fungus back into a live host for continued *in vivo* host to host maintenance of *E. muscae* culture, and thus not for carrying out injection-based infection assays (Fig. 3). This procedure may alter the usual temporal restrictions of the fungal infection, i.e. the flies may not die exactly 6–7 days post injection and may not display the characteristic behavioural manipulation seen in *E. muscae* infections. Why these changes occur is unknown, however the stereotypical infection is usually resumed in the next round of infections using the sporulating cadavers resulting from infection by injection.

### Materials

• Live healthy adult houseflies.
• CO_2_ (carbon dioxide).
• 10 µL micro-syringe (e.g. Kloehn CO., INC, Whittier, California, U.S.A.) (see Fig. 3).
• 1–3 µL of liquid fungal culture per fly.
• 1.5 mL Eppendorf.
• Centrifuge.
• 1000 mL range sterile pipette.
• Sterile cut-off 1 mL pipette tip (use a pair of scissors to cut off the tip to widen the entrance hole and sterilise in autoclave).
• Housefly housing container (as mentioned *in vivo* maintenance section above).
  1. Use a sterile 1 mL cut-off pipette tip and place 500–800 µL of actively growing *E. muscae* culture in 1.5 mL Eppendorf.
  2. *Optional, but recommended*: Gently spin down the fungal culture in centrifuge at low speed (<200 rcf, 5–10 min) to not kill the fungal cells.
  3. *Optional, but recommended*: Carefully remove some of the supernatant media to concentrate the fungal cells.
  4. Using CO_2_ (carbon dioxide) anaesthetised houseflies, hold the fly firmly in one hand.
  5. Using a micro-syringe, gently pierce the side of the thorax of the restrained fly.
  6. Inject 1–3 µL amount of concentrated liquid fungal culture and place housefly in the usual housefly cage setup (Fig. 3).
  7. Allow 3 to 14 days for the infection to kill the flies and sporulate from the abdomen as normal. With this technique, at least 10% of the flies develop infection. Usually more, but depends on the virulence and state of the fungal culture used for infection.
  8. Use these flies to continue the *in vivo* infection as per the previous protocol.

## Controlled *E. muscae* infections for virulence experiments

**Fig. 4:**
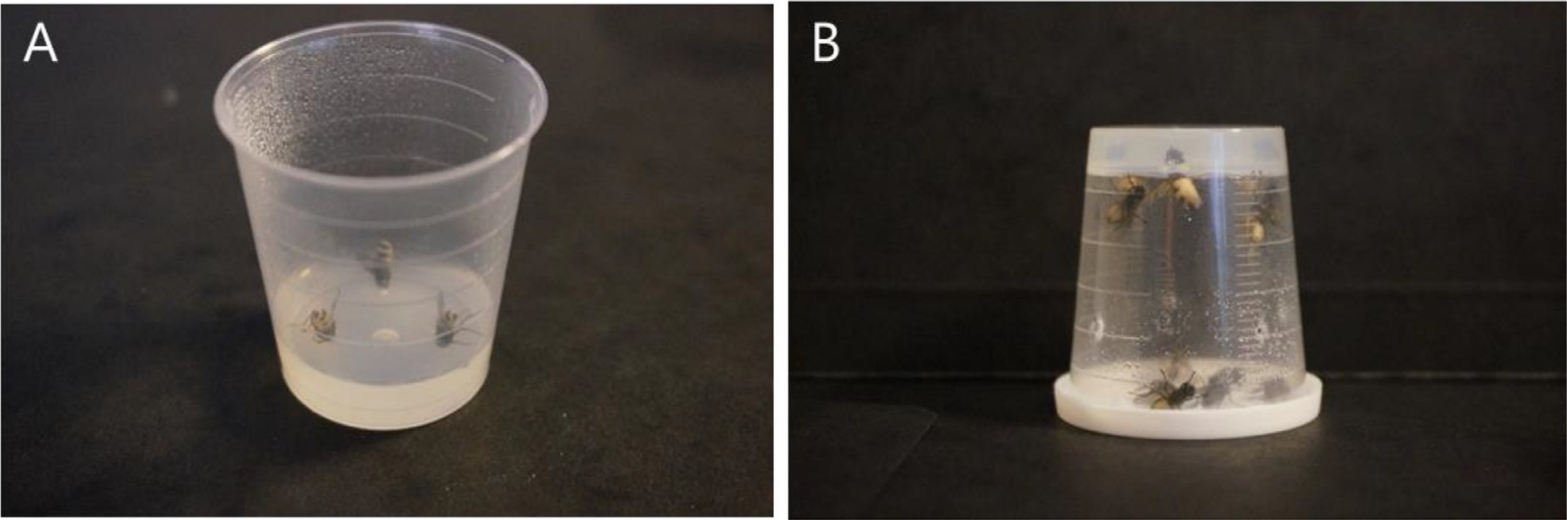
*In vivo* exposure setup for experiments (A) Three cadavers are placed head first into water agar with abdomens exposed for *in vivo* infections. (B) The medicine cup containing the cadavers and the uninfected flies is then turned upside down and placed into a humid chamber for 24 h. Photos: S. Edwards.

This protocol is used for experiments that need a guaranteed exposure to *E. muscae* conidia and death from infection 6 or 7 days post exposure to infected cadavers (Fig. 4). The high exposure rate to conidial showers provide near 100% death by day 6 in our system, with deaths prior to day 6 not being caused by the fungus as there is no behavioural manipulation and no fungal sporulation from the cadavers.

### Materials

• Three dead sporulating cadavers (from the same fungal isolate) – with 2:1 or 1:2 male:female cadaver sex ratio to account for sporulation differences in cadaver sex.
• Medicine cup containing 2–5 mL of 1.5% water agar.
• Entomology forceps.
• CO_2_ (carbon dioxide) or cold room at ∼5 °C.
• Humid chamber (as mentioned *in vivo* maintenance section above).
• 18–23 °C incubator on light:dark cycle between 16:8 and 12:12 (keep constant during experiments).
• Housefly housing container (as mentioned *in vivo* maintenance section above)
  1. Using cleaned forceps, gently grab a cadaver by the head and thorax and bury the head into the agar, keeping the abdomen exposed. Making a hole in the agar beforehand will reduce the risk of decapitating the cadaver. For mock-infections for an uninfected control treatment, replace the sporulating cadavers by freeze-killed flies (freeze-kill with 5–10 min exposure to −5 or −20) (Fig. 4A).
  2. Perforate the lid and cup sides to allow for aeration during infection.
  3. After placing three cadavers in this manner, add up to 10 anaesthetised (CO_2_ or cold exposure depending on experiments) flies and place the medicine cup upside down in the humid chamber for 24 h (as little as six hours exposure has also worked in our laboratory with near 100% infection) (Fig. 4B).
  4. After 24 h, remove the live flies and place in a normal housing container. Discard the medicine cup and cadavers.
  5. Keep the flies at constant light:dark cycle and temperature for accurate planning of manipulation behaviours and death.

## Quantification of *E. muscae* conidia discharge per cadaver

**Fig. 5:**
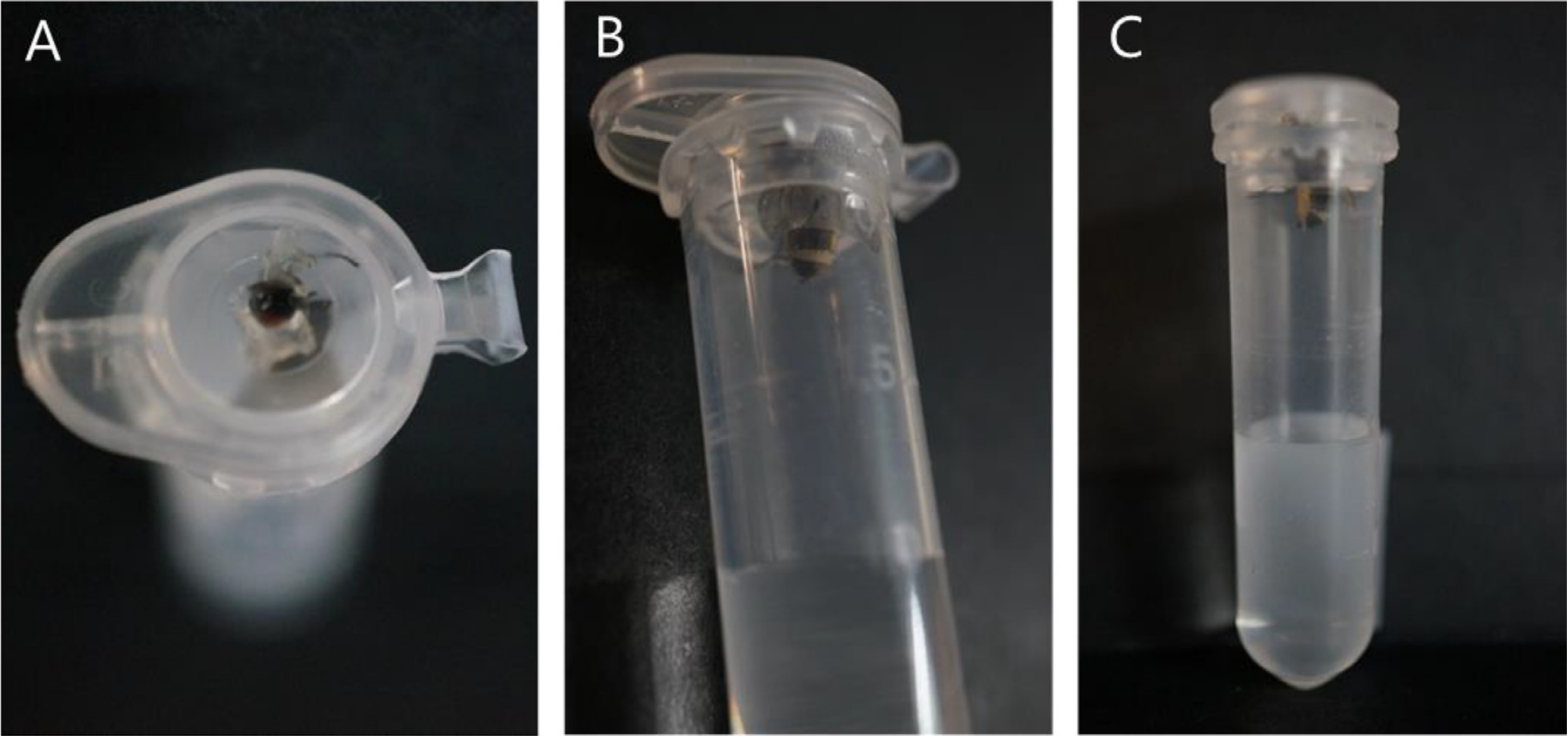
Quantifying conidial discharge from *E. muscae* sporulating housefly cadavers. (A) Cadavers are placed head first into vaseline® in the eppendorf cap (B) with abdomens exposed. (C) The conidia are collected in the 1% Triton-X and 0.2% maleic acid solution. Photos: S. Edwards.

This protocol is used to calculate the exposure dosage of *E. muscae* from individual sporulating cadavers (Fig. 5). Variations can be found in different host species and *E. muscae* isolates, making this a simple protocol for checking the discharge dosage. The *E. muscae* conidia are collected in an acid solution to prevent germination of discharged conidia [25]. These can be counted in a haemocytometer under a microscope or using image analysis [30]. See Figs. 3&4 in [21] and Figs. 7&8 in [7] for examples of microscope images of conidia.

### Materials

• Dead sporulating cadavers – variations per individual cadaver are high and it should therefore be considered to repeat this with both male and females to account for sporulation differences of cadaver sex.
• 2 mL Eppendorf tube.
• 1% Triton-X.
• 0.2% maleic acid.
• Vaseline® (Conopco, Inc., USA).
• 0.2 mm haemocytometer (e.g. Fuchs-Rosenthal Chamber, 3720).
• Entomology forceps
  1. Add Vaseline inside the lid.
  2. Prepare a solution containing 1% Triton-X and 0.2% maleic acid.
  3. Place 1 mL of the above solution in an Eppendorf.
  4. Using cleaned forceps, gently grab a cadaver by the head and thorax and bury the head into the Vaseline, keeping the abdomen exposed.
  5. Leave to sporulate for duration of interest. For example, for 24 h if wanting to quantify the conidial discharge under conditions in above protocol “Controlled *E. muscae* infections for virulence experiments”.
  6. After selected duration, add solution to a haemocytometer and place under a microscope for determining spore concentration.

## Ethics statements

We have used fungi and houseflies for our experiments, all of which complied with our institution's safety and laboratory guidelines.

## CRediT authorship contribution statement

**Sam Edwards:** Project administration, Conceptualization, Methodology, Investigation, Validation, Writing – original draft, Writing – review & editing. **Henrik H. De Fine Licht:** Supervision, Funding acquisition, Project administration, Resources, Conceptualization, Methodology, Writing – original draft, Writing – review & editing.

## Declaration of Competing Interest

The authors declare that they have no known competing financial interests or personal relationships that could have appeared to influence the work reported in this paper.

## Data Availability

No data was used for the research described in the article.
